# Ongoing Dynamics of Peak Alpha Frequency Characterize Hypnotic Induction in Highly Hypnotic-Susceptible Individuals

**DOI:** 10.3390/brainsci14090883

**Published:** 2024-08-30

**Authors:** Mathieu Landry, Jason da Silva Castanheira, Floriane Rousseaux, Pierre Rainville, David Ogez, Karim Jerbi

**Affiliations:** 1 Département de Psychologie, Université du Québec à Trois-Rivières, Trois-Rivières, QC G9A 5H7, Canada; 2 Department of Experimental Psychology, University College London, London WC1E 6BT, UK; jason.dasilvacastanheira@mail.mcgill.ca; 3 Centre de Recherche Hôpital Maisonneuve-Rosemont, Montreal, QC H1T 2M4, Canada; floriane.rousseaux@umontreal.ca (F.R.); david.ogez@umontreal.ca (D.O.); 4 Départment de Stomatologie, Faculté de Médecine Dentaire, Université de Montréal, Montréal, QC H3T 1J4, Canada; pierre.rainville@umontreal.ca; 5 Centre de Recherche de l’Institut Universitaire de Gériatrie de Montréal (CRIUGM), Université de Montréal, Montréal, QC H3W 1W6, Canada; 6 Département d’Anesthésiologie et de Médecine de la Douleur, Université de Montréal, Montreal, QC H3C 3J7, Canada; 7 Département de Psychologie, Université de Montréal, Montreal, QC H3C 3J7, Canada; karim.jerbi@umontreal.ca; 8 MILA-Quebec Artificial Intelligence Institute, Montreal, QC H2S 3H1, Canada; 9 UNIQUE Center (Quebec Neuro-AI Research Center), Montreal, QC H3T 1P1, Canada

**Keywords:** hypnosis, resting-state EEG, spectral analysis, aperiodic activity, alpha frequency, machine learning, altered state of consciousness, classification, arrhythmic activity

## Abstract

Hypnotic phenomena exhibit significant inter-individual variability, with some individuals consistently demonstrating efficient responses to hypnotic suggestions, while others show limited susceptibility. Recent neurophysiological studies have added to a growing body of research that shows variability in hypnotic susceptibility is linked to distinct neural characteristics. Building on this foundation, our previous work identified that individuals with high and low hypnotic susceptibility can be differentiated based on the arrhythmic activity observed in resting-state electrophysiology (rs-EEG) outside of hypnosis. However, because previous work has largely focused on mean spectral characteristics, our understanding of the variability over time of these features, and how they relate to hypnotic susceptibility, is still limited. Here we address this gap using a time-resolved assessment of rhythmic alpha peaks and arrhythmic components of the EEG spectrum both prior to and following hypnotic induction. Using multivariate pattern classification, we investigated whether these neural features differ between individuals with high and low susceptibility to hypnosis. Specifically, we used multivariate pattern classification to investigate whether these non-stationary neural features could distinguish between individuals with high and low susceptibility to hypnosis before and after a hypnotic induction. Our analytical approach focused on time-resolved spectral decomposition to capture the intricate dynamics of neural oscillations and their non-oscillatory counterpart, as well as Lempel–Ziv complexity. Our results show that variations in the alpha center frequency are indicative of hypnotic susceptibility, but this discrimination is only evident during hypnosis. Highly hypnotic-susceptible individuals exhibit higher variability in alpha peak center frequency. These findings underscore how dynamic changes in neural states related to alpha peak frequency represent a central neurophysiological feature of hypnosis and hypnotic susceptibility.

## 1. Introduction

Hypnosis involves guiding individuals into a focused mental state that often results in altered conscious experiences and heightened responsiveness to suggestions [1,2]. Notably, individuals’ capacity to respond to hypnotic suggestions varies widely [3,4]. While some find it challenging to achieve the desired outcomes, others are capable of significant changes in perception, cognition, and emotion due to these suggestions [5,6]. Highly hypnotic-susceptible individuals (HHSIs), in particular, can exert extraordinary control over mental processes that are typically difficult to regulate [7]. Researchers assess this variability in susceptibility using standardized scales that have demonstrated high test–retest reliability in adulthood, underscoring the stability of hypnotic susceptibility over time [8,9,10,11]. The distribution of scores from these assessments tends to be normal, with most individuals falling within the moderate range, and those at the extremes—both low and high susceptibility—comprising about 20–30% of the population [12]. Understanding the variability in hypnotic susceptibility represents a central goal for research on hypnotic phenomena, as it may inform personalized approaches to hypnosis-based therapies and enhance our comprehension of the psychological mechanisms underlying hypnosis.

Significant research efforts have gone into uncovering the latent factors underlying inter-individual differences in hypnotic responding [13]. Rather than attributing susceptibility to hypnotic suggestions to a single such component, evidence relates it to multiple ones. In fact, several models tend to highlight that the psychological structure of hypnotic responding rests on a general overarching factor supported by several secondary ones [10,14,15]. These findings underscore the centrality of motor-challenging suggestions (i.e., suggestions aimed at incapacitating movements) for determining one’s susceptibility to hypnotic suggestions [16]. Furthermore, in the context of these motor-challenging suggestions, HHSIs are more likely than low hypnotic-susceptible individuals (LHSIs) to feel incapable of performing the suggested movement and to perceive this inability as occurring independently of their own volition [17]. This is consistent with observations showing that a reduced sense of agency represents a key feature of hypnotic responding, at least for some individuals [18,19]. Although the reasons why motor-challenging suggestions are central to explaining hypnotic susceptibility remain unclear, their centrality implies that effective responses to hypnotic suggestions involve phenomenological distortions of motor control [20], while neurophysiological evidence links these phenomena to brain regions involved in executive control and monitoring [21].

At the neural level, studies have linked hypnotic susceptibility scores to both structural and functional neural patterns [22,23]. At the structural level, evidence suggests that hypnotic susceptibility is associated with variability in both white and gray matter. However, it is important to note that these findings require further replication to confirm their validity. At the functional level, reports link modulations of connectivity patterns across the default, saliency, central executive, and dorsal attention networks to a wide range of hypnotic phenomena. Recently, our work used multivariate pattern classification to investigate whether features derived from spectral analysis and connectivity patterns from resting-state electroencephalography (EEG) could distinguish between HHSIs and LHSIs based on neural activity recorded both at baseline and following hypnotic induction (outside and inside of hypnosis, respectively [24]). Multiple neural features across both recording conditions discriminated between HHSIs and LHSIs. This outcome aligns with previous work showing that susceptibility to hypnotic suggestion involves multiple neural components, rather than a single one [25]. Importantly, this work reveals that EEG arrhythmic activity recorded outside of hypnosis represents the top neural feature to discriminate between HHSIs and LHSIs. This outcome supports the idea that susceptibility to hypnotic suggestion represents a latent trait and underscores ongoing research efforts to develop assessment tools for hypnotic susceptibility grounded in neurophysiological recordings [26]. These advances also highlight the complex and multifaceted nature of hypnotic susceptibility, paving the way for more personalized and effective applications of hypnosis in clinical and research settings.

Several studies link hypnosis to modulations of spectral power within alpha and theta rhythms [27]. More specifically, while evidence relating alpha-band activity to hypnosis varies across assays, the most consistent finding relates increased theta-band power to hypnosis. However, the exact relationship between alpha and theta rhythms and hypnotic phenomena remains elusive. One hypothesis links the modulation of slower brain rhythms to the engagement of declarative memory systems during hypnosis, as these systems are recruited by verbal instructions and suggestions during this process [28]. Contrary to these findings, however, our recent work challenges the pivotal role traditionally attributed to these neural components in hypnotic phenomena [24]. In this work, neither rhythmic alpha nor theta spectral power was a key feature in distinguishing between HHSIs and LHSIs, either before or after the induction process. A critical methodological difference that might explain this discrepancy is our control for the arrhythmic component of the power spectrum prior to analyzing theta-band and alpha-band power. This adjustment casts doubt on the significance of these spectral changes when arrhythmic activity is considered, suggesting that previous interpretations may need re-evaluation.

**Figure 1:**
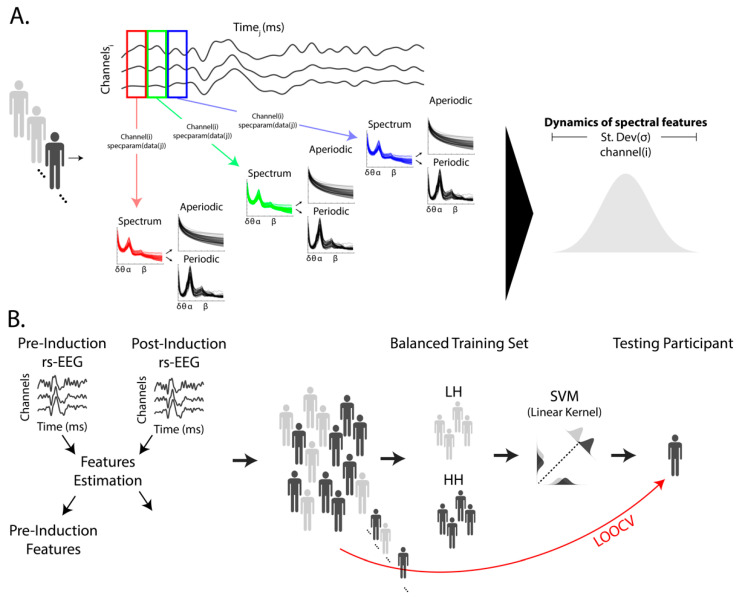
(**A**) Our analysis pipeline employed the SPRiNT algorithm to extract the variability in rhythmic component parameters in alpha-band activity (8 to 13 Hz) and the parameters of the arrhythmic component using 5 min of eye-closed resting-state EEG (rs-EEG) data, both pre- and post-hypnotic induction. SPRiNT, a time-resolved implementation of the FOOOF algorithm via specparam in MATLAB, was used to analyze each one-second time window, extracting the relative amplitude, width, and center frequency of the peak within the alpha-band range for each rhythmic component, as well as the offset and exponent of the arrhythmic component for each individual and channel i. The standard deviation across the time series was computed to estimate the variability in each parameter to evaluate the dynamics of spectral features (**B**). We extracted these estimates separately for the pre- and post-induction periods and evaluated the effects for the pre-induction period compared to Δinduction (values from post-induction minus values from pre-induction). Individuals with high and low hypnotic susceptibility were classified based on these metrics using multivariate pattern analysis with leave-one-out cross-validation (LOOCV) employing a linear support vector machine (SVM). EEG channels served as features for this linear model. Each training iteration included balanced classes of individuals with low (light grey) and high (dark grey) hypnotic susceptibility, with one participant left out for validation.

Instead, our previous work shows that the hypnotic induction differentially affects delta-band spectral power and connectivity patterns across delta, theta, alpha, and beta frequency bands between HHSIs and LHSIs. Still, the extent to which these neural patterns facilitate the emergence of hypnotic phenomena and responses remains uncertain and necessitates further corroboration. One limitation of the analyses from our previous work is that we focused on time-averaged values for each neural feature between HHSIs and LHSIs, both outside and inside hypnosis. This approach assumes stationarity and overlooks the dynamics of these neural features—specifically, the degree to which they vary during EEG recordings. The current study addresses this lacuna by examining the variability in spectral features during EEG recordings, comparing how these variations differ between HHSIs and LHSIs both in and out of hypnosis. Specifically, we employed time-resolved spectral decomposition to dissect the rhythmic and arrhythmic components of resting-state EEG activity (Figure 1A, [29]), focusing on the temporal variations of these features. Thus, our methodology centers on the ongoing dynamics of neural patterns as a function of hypnotic susceptibility and hypnotic induction, offering a deeper understanding of the underlying neural mechanisms.

Specifically, the present study examines variations along the time series of five distinct neural features of resting-state EEG signals, which can be broadly categorized into oscillatory (rhythmic) and non-oscillatory (arrhythmic) components. Oscillatory or periodic activity reflects rhythmic fluctuations that manifests prominently as peak amplitude within a range of frequency bands of the EEG signal’s spectral decomposition. Most notably, the human EEG is characterized by a peak amplitude occurring approximately within the range of alpha rhythms, between 8 and 13 hz—a phenomenon first documented in the earliest EEG recordings of the human brain [30]. Interestingly, individual peak alpha frequency (IAPF) correlates with diverse psychological domains such as perception (e.g., [31]), cognition (e.g., [32]), developmental (e.g., [33]), aging (e.g., [34]), cognitive decline (e.g., [35]) and psychopathology (e.g., [36]). The IAPF is thus considered a stable neurobiological marker of psychological functioning [37]. In turn, non-oscillatory or arrhythmic activity corresponds to arrhythmic EEG activity which approximates a 1/f-like power-law function in the spectral domain [38]. Mounting evidence emphasizes the importance of considering both oscillatory and non-oscillatory components to fully understand the neural underpinnings of human cognition [39]. Our latest findings extend this perspective, linking the exponent of non-oscillatory EEG activity to hypnotic susceptibility even outside hypnotic states, reinforcing the significance of these spectral components in cognitive neuroscience [24].

The goal of the present work was to explore the ongoing dynamics of EEG spectral features during EEG recordings as a function of hypnotic susceptibility and hypnotic induction. In this effort, we assessed the ongoing variability in rhythmic and arrhythmic spectral features of the EEG.

In this effort, we investigated whether variations in non-oscillatory and oscillatory activities during resting-state EEG could differentiate between HHSIs and LHSIs, both outside and inside of hypnosis, i.e., before and after hypnotic induction. To test this, we utilized time-resolved parametrization of the power spectrum, extracting variations in offset and exponent estimates of the 1/f-like power-law function across the time series for each EEG channel and participant [29]. Additionally, given the established relationship between alpha rhythms and hypnosis (for review, see [27]), we also examined variations in alpha peaks. In the context of spectral decomposition, peaks occurring at specific frequencies can be modelled as a Gaussian function characterized by three parameters: amplitude relative to the non-oscillatory 1/f-like activity, peak width, and the center frequency [38]. We accordingly investigated these parameters relative to alpha-band activity. Moreover, our approach also included multivariate pattern classification where we trained and tested a linear classifier to distinguish between HHSIs and LHSIs based on topographical patterns. This approach enhances the detection capabilities compared to univariate approaches [40], potentially providing a more robust analysis of the neural correlates of hypnotic susceptibility. Consistent with an extensive body of research [27], our study aimed at distinguishing between HHSIs and LHSIs. This approach provides a proof of concept based on a two-class solution for identifying specific neural features that could predict participants’ levels of hypnotic susceptibility.

## 2. Methods

### 2.1. Participants

Participants were initially invited to a pre-screening session where their hypnotic susceptibility was assessed using the Harvard Group Scale of Hypnotic Susceptibility, Form A (HGSHS:A; [41]). From this initial group of participants, 75 individuals were selected for further EEG recordings. For the present study, we focused on neural activity data from 40 individuals (27 females, mean age 21.98 years, SD = 4.41) who scored above 8, indicating HHSIs, and those who scored below 4, indicating LHSIs. This selection aligns with previous studies that have focused on individuals with high and low hypnotic susceptibility to enhance the detection of effects. Participants were not informed of their scores. Those who completed the second part of our recruitment procedure received monetary compensation. All study procedures were approved by the Faculty of Medicine Institutional Review Board at McGill University (certificate number A03-B56-17B), and participants provided written informed consent.

The same dataset was previously utilized to explore spectral features and graph theory metrics derived from connectivity patterns [24]. In this previous study, we explained how our sample size was based on earlier neurophysiological investigations of hypnotic susceptibility. In the present study, however, two participants—one individual with high hypnotic susceptibility and one individual with low hypnotic susceptibility—were excluded because the SPRiNT algorithm failed to model alpha peaks in certain channels for these individuals.

### 2.2. Procedure

The assessment of hypnotic susceptibility was conducted according to the standard HGSHS:A protocol during a separate experimental session, held on a different day from the EEG recordings. Following the assessment of hypnotic susceptibility, the EEG recording sessions included two resting-state periods: an initial 5 min recording without hypnosis (pre-induction period), followed by a hypnotic induction, and then another 5 min recording (post-induction period). For further details on the induction script, refer to the Supplementary Materials.

During the EEG recordings, participants were seated in a quiet, dimly lit room. The experimenter, who also conducted the induction, was positioned approximately one meter behind the participants throughout the session. To prevent potential contamination (i.e., carry-over effects) from the hypnotic induction, we recorded the baseline EEG for all participants before the induction-related EEG. This precaution was necessary due to the indeterminate duration required for the effects of hypnosis to dissipate after de-induction. Given that the primary objective of the study was to differentiate between individuals with high and low hypnotic susceptibility, the sequence of baseline and induction-related EEG recordings was considered irrelevant. During the baseline recording, participants were instructed to close their eyes while remaining alert. For the induction period, the experimenter read from a script (detailed in the Supplementary Materials) that included instructions for participants to close their eyes. The 5 min post-induction EEG was recorded immediately following the induction script’s completion. The induction procedure was conducted by experimenters who had previously been trained in hypnosis. Experimenters were aware of participant’s level of hypnotic susceptibility.

### 2.3. Electroencephalography

EEG signals were recorded using 64 Ag/AgCl active electrodes (ActiCap System; Brain Products GmbH; Gilching, Germany), while all electrophysiological signals were amplified using the ActiChamp System (Brain Products GmbH; Gilching, Germany). We added bipolar electrodes at the outer canthi and the superior and inferior orbits of the left eye. Impedances of all electrodes were kept below 10 kΩ. EEG data were initially recorded at a sampling rate of 1000 Hz, then downsampled to 250 Hz offline for analysis. Data acquisition was facilitated using the Brain Recorder software (Brain Products Inc., GmbH, Gilching, Germany). Subsequent preprocessing and data analysis were conducted using Brain Vision Analyzer, Brainstorm [42], and custom MATLAB scripts (version R2022A; MathWorks Inc., Natick, MA, USA). Any faulty electrodes were topographically interpolated using spherical splines, affecting less than 3% of electrodes overall. EEG recordings were re-referenced offline to the average of all EEG electrodes to ensure consistency. All datasets were rigorously inspected for muscle-related, eye movement, or technical artifacts, which were meticulously removed prior to analysis.

### 2.4. Spectral Features

Spectral features were computed individually for each channel using the Spectral Parametrization Resolved in Time (SPRiNT) algorithm for spectral parametrization as implemented in Brainstorm (Figure 1A [29,42]). The SPRiNT algorithm implements a time-resolved approach to the Fitting Oscillations and One-Over-f (FOOOF; also known as *specparam*) algorithm, which decomposes power spectra into rhythmic and arrhythmic components by parameterizing the power spectral density in log-log space [38]. This transformation linearizes the 1/f component, facilitating its separation from oscillatory components. In this way, the arrhythmic component is defined by the offset and exponent of the power spectrum density, whereas the rhythmic component is characterized by the center frequency, bandwidth, and relative amplitude of the oscillatory peak. Specifically, in the context of the FOOOF algorithm, the transformation of the power spectrum in log-log space is defined as the following linear combination:

(1) $$L+\sum_{n=0}^{N}G_{n}$$

where *L*, the arrhythmic component, is modelled as a Lorentzian function:

(2) $$L=b-log(F^{x})$$

where *b* corresponds to the broadband offset, *x* is the exponent or slope, and *F* represents an input vector of frequencies. Note that we did not model a knee in the arrhythmic component. In turn, each rhythmic peak *n* of the power spectrum is defined as a Gaussian function *G*:

(3) $$G_{n}=a\times exp\left(\frac{{-(F-c)}^{2}}{{2w}^{2}}\right)$$

where *a* represents the relative amplitude in log-log space, *c* corresponds to the center frequency in Hz, *w* is the standard deviation of the Gaussian function (i.e., width), and *F* is an input vector of frequencies.

The SPRiNT algorithm utilizes the FOOOF parameterization [38] technique on neural time series through overlapping time windows. The length of these windows for applying the short-time Fourier transform was set to 1 s, with a 50% overlap between sliding windows. The power spectral density ranged from 1 to 40 Hz. We evaluated up to three peaks, with parameters set as follows: a minimum peak height of 1 dB, a peak threshold of 2, a proximity threshold of 0.75, peak width limits between 1.5 and 6 Hz, and a fixed arrhythmic mode. These values were determined based on previous work using SPRiNT to avoid overfitting the power spectrum [29]. Goodness of fit was assessed based on mean-square error (MSE). Goodness-of-fit values were acceptable with more than 80% of all electrodes across all participants showing a MSE > 0.8 during both pre-induction and post-induction periods. Importantly, no significant differences were observed between individuals of high and low hypnotic susceptibility during the pre-induction period and Δinduction (post-induction minus pre-induction). Specifically, the area under the curve (AUC) for classification was 0.53 (*p* = 0.11) for pre-induction EEG and 0.43 (*p* > 0.9) for Δinduction on EEG, using the multivariate pattern classification described below. Likewise, variability in mean-square error across the time series also failed to predict individuals of high and low hypnotic susceptibility during the pre-induction period and Δinduction (values from post-induction minus values from pre-induction).

For each one-second time window, we extracted parameters for the relative amplitude, peak width, and center frequency of alpha-band activity (8 to 13 Hz), along with the offset and exponent of the arrhythmic component. We examined the dynamic patterns of the spectral features based on the standard deviation of each parameter across the time series (Figure 1A). To assess the neural dynamics related to hypnotic susceptibility, we calculated the variability for each electrode during the pre-induction period and Δinduction (values from post-induction minus values from pre-induction) using this approach.

### 2.5. Multivariate Pattern Analysis and Leave-One-Out Cross-Validation

We employed a multivariate pattern analysis using a linear support vector machine (SVM) model, coupled with a leave-one-out cross-validation strategy. The goal was to differentiate between HHSIs and LHSIs based on parametrization of the power spectrum using parameter estimates of the exponent and offset for the arrhythmic component, as well as the width, relative amplitude and center frequency for the alpha peak. These parameters were estimated separately for each channel and participant for pre-induction resting-state EEG and Δinduction (values from post-induction minus values from pre-induction). Classification performance between HHSIs and LHSIs was evaluated for each of these parameters separately across both the pre-induction and Δinduction. Hence, for each parameter (i.e., exponent, offset, width, amplitude, and center frequency), our SVM model was trained to separate HHSIs and LHSIs based on data from 64 EEG channels as predictors. To minimize classification biases and maintain class balance [43], our training sets in each iteration included 15 HHSIs and 15 LHSIs. Next, the cross-validation procedure was performed by evaluating whether the model that was trained could accurately predict the class (HHSIs or LHSIs) of a participant that was left out of the training procedure. Each participant was selected once per iteration for this evaluation procedure, while 15 participants from each susceptibility class were randomly selected for the training set. In sum, we excluded a participant from the training set, then randomly selected 15 HHSIs and 15 LHSIs for training the SVM model, and lastly evaluated the classifier’s ability to accurately predict the class (HHSIs or LHSIs) of the excluded participant. This procedure was repeated 10 times, providing robust cross-validation coverage.

Given our balanced training set, model performance was assessed using the area under the curve (AUC) metric. The classification performance was benchmarked against a surrogate distribution derived from 1000 random permutations, where class labels were randomly shuffled during the training process [44]. A performance was deemed statistically significant if it exceeded the 95th percentile of this surrogate distribution, with significance set at α = 0.05 for one-tailed assessment. We applied Bonferroni correction to account for multiple tests.

To aid in interpreting the SVM classifiers, we transformed the weights based on the covariance of the training dataset [45]. These transformed weights were subsequently averaged across all training iterations. We evaluated SVM features separately for the pre-induction phase and changes post-induction.

### 2.6. Complexity

Complexity estimates the degree of intricacy and variability within a signal, including the amount of information conveyed through structural patterns and randomness [46]. Moreover, previous research indicates that complexity measures are associated with hypnotic susceptibility [47]. Based on these insights, we employed the Lempel–Ziv complexity measure, a method for quantifying the complexity of a time series by counting the number of distinct patterns it contains [48].

We calculated the Lempel–Ziv complexity for every EEG channel and each participant by first segmenting the EEG time series data into 1 min epochs. In each epoch, we converted the time series into binary sequences of 0 s and 1 s. This binary transformation was achieved by comparing each EEG value to a predefined threshold: values below the threshold were assigned a ‘0’, and those above were assigned a ‘1’. We then applied the Lempel–Ziv complexity algorithm using an exhaustive method to enumerate all unique patterns or substrings in the binary sequences from each epoch and channel. This analysis was conducted using a publicly available MATLAB script from the MATLAB Central File Exchange [49]. Subsequently, we implemented a multivariate classification strategy to analyze the data from pre-induction and Δinduction periods separately.

## 3. Results

**Figure 2:**
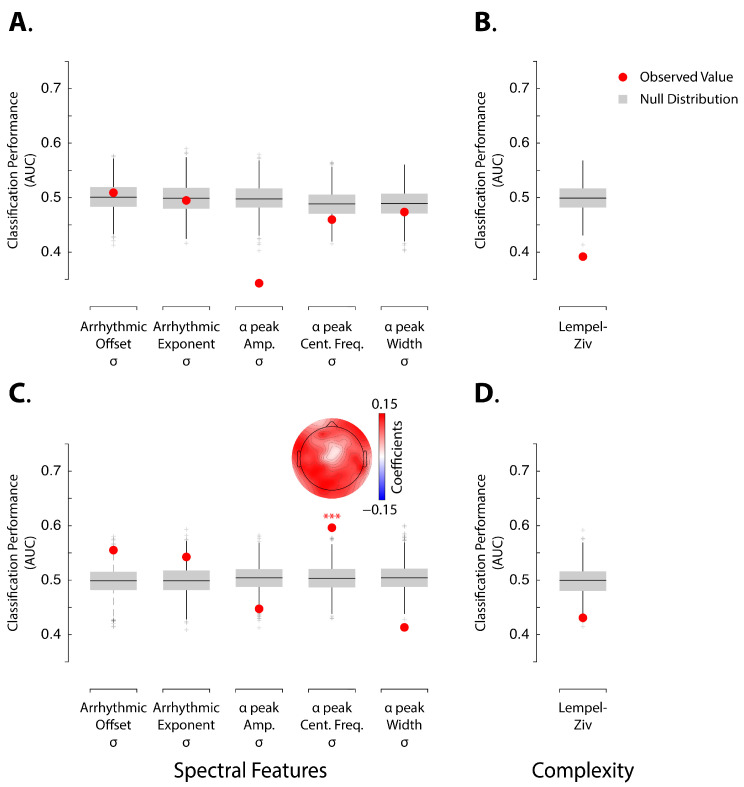
Panels (**A**,**B**) show analysis for pre-induction EEG, whereas panels (**C**,**D**) show analysis for Δinduction EEG. Panels (**A**–**D**) show AUC values for assessing classification performances of SVM linear models for accurately classifying high and low hypnotic-susceptible individuals in LOOVC based on arrhythmic and alpha-band related features of the power spectrum during the EEG pre-induction period (**A**) and the Δinduction period (**C**), as well as Lempel–Ziv complexity coefficients calculated during the EEG pre-induction period (**B**) and for Δinduction (**D**). Red dots show observed AUC values. Boxplot shows surrogate null distributions based on random permutations where we shuffled the labels during training. The bottom and top whiskers indicate the first and third quartiles, respectively. Three asterisks indicate statistical significance at *p* < 0.001. Topographies show the averaged coefficient values of the SVM models from all iterations and LOOVC approach for the neural features that can discriminate between high and low hypnotic-susceptible individuals better than chance-level.

We examined the ongoing dynamics based on the variability of spectral features along the time series (Figure 2). For the pre-induction resting-state period, none of the spectral features reliably distinguished between HHSIs and LHSIs (Figure 2A; all *p*-values > 0.3). In contrast, for Δinduction, variations in the alpha peak frequency significantly discriminated between HHSIs and LHSIs (Figure 2C; Area Under the Curve [AUC] = 0.6, *p* < 0.001). However, classifications based on all other features during Δinduction did not achieve significant discrimination of LHSIs (Figure 2C). Notably, during Δinduction, the variability of the arrhythmic offset (Figure 2C; AUC = 0.56; *p*-value = 0.02) and the arrhythmic exponent (Figure 2C; AUC = 0.54; *p*-value = 0.05) showed high classification values relative to the null distribution, but these did not survive correction for multiple comparisons.

Our results show that variations in alpha center frequency distinguish between HHSIs and LHSIs above chance level following the induction procedure. This outcome indicates that the dynamics of brain states, as reflected by alpha center frequency activity, represents a key neurophysiological feature of hypnotic phenomena. Alongside this finding, we investigated whether EEG signal complexity could also differentiate HHSIs from LHSIs. Classification based on Lempel–Ziv complexity did not achieve significant discrimination of LHSIs, neither during the pre-induction resting-state period nor the Δinduction phase (Figure 2B,D).

## 4. Discussion

Using multivariate pattern classification, this study investigated whether temporal variations in arrhythmic and rhythmic alpha-band activity change as a function of hypnotic susceptibility and whether it is measured prior to or following a hypnotic induction. Prior research highlights the importance of neural oscillations in studying hypnotic phenomena [50,51]. A previous study by our group extends this line of research by demonstrating that non-oscillatory EEG activity also plays a significant role in hypnotic phenomena [24]. Specifically, in that study, we found that the exponent of the arrhythmic component represents a key neuronal feature for distinguishing between HHSIs and LHSIs outside of hypnosis. This finding implies that hypnotic susceptibility corresponds to a latent neural trait. The present work extends previous observations by exploring if variability in the arrhythmic and rhythmic components along the time series recorded inside and outside of hypnosis would similarly differentiate between HHSIs and LHSIs. This exploratory approach revealed that significant variations in the alpha peak frequency following the induction procedure are indicative of hypnotic susceptibility (Figure 2C). This was the sole significant outcome we observed in our analysis after correcting for multiple comparisons. Our finding provides further illumination into the neurobiological substrates of susceptibility to hypnosis and helps identify biomarkers that predict hypnotic responding [23]. Although our observation does not directly provide insights into the underlying mental states or cognitive processes involved in the induction procedure, this outcome aligns with the broader principles of precision medicine, particularly in predicting real-world outcomes based on neural features [52,53]. Here, we demonstrate the possibility of using the resting-state EEG in relation to the induction procedure to differentiate LHSIs from HHSIs.

Specifically, we observed notable differences in the variability of alpha peak center frequency during hypnosis, with HHSIs exhibiting greater variations compared to LHSIs. Alpha peak frequency closely relates to overall brain states [37]. In this regard, the topographical pattern of the modified SVM weights shows that greater variability in IAPFs for HHSIs following the induction was widespread across the cortex, rather than localized to a specific cortical region. This outcome implies that hypnotic phenomena do not rest on a static brain pattern and instead appear to enhance the overall dynamic fluctuations of neural states for HHSIs. Furthermore, our result aligns with and extends recent research by Rho et al. [47], who employed the Lempel–Ziv algorithm to reveal greater complexity in the EEG alpha frequency signals of HHSIs compared to LHSIs following hypnotic induction. In the context of EEG, complexity refers to the regularity and predictability of brain patterns where increased complexity entails greater intricacy and variability of the EEG waveform patterns. The observation of Rho et al. [47] therefore reveals increased variability in neural patterns in alpha rhythms among HHSIs as a function of the induction. Insofar that alpha peak frequency is indicative of overarching brain states, our results are consistent with this observation. It is important to note, however, that while Rho et al. [47] measured complexity in terms of the dynamics of amplitude within the alpha-band, our study focuses on the center frequency of the peak in the alpha range. Moreover, we further expanded our investigation to determine whether the complexity of the broadband EEG signal beyond alpha patterns is integral to understanding hypnotic susceptibility and induction. However, our findings do not support the notion that overall EEG signal complexity is a key characteristic of hypnotic susceptibility. This suggests that the observed variability in neural states may be specific to alpha oscillations.

Our findings hint that hypnosis may enhance neural adaptability, potentially indicative of increased response preparation. Indeed, the process of formal hypnotic induction is often designed to facilitate heightened readiness to respond [54]. Most notably, the induction includes a procedure identification step, where individuals are informed about the upcoming hypnosis session, which likely increases participants’ expectations about their experience and influences their response to forthcoming suggestions [55]. This particular aspect shares similarities with the placebo responses [56]. Additionally, the induction process includes targeted instructions that further reinforce these expectations, thereby bolstering preparatory activity [57]. As a result, the increased variability observed in alpha-band neural patterns may be a marker of this enhanced preparatory state. The hypnotic induction is also characterized by spontaneous phenomenological changes, such as heightened feelings of relaxation, mental absorption, a sense of dissociation, and temporal distortions [58], which our findings may also reflect. However, it is noteworthy that previous EEG studies have not consistently linked alpha-band activity with the spontaneous phenomenological changes experienced during hypnosis as a function of hypnotic susceptibility [59]. Furthermore, the IAPF relates to various aspects of perception and cognition [31,60], including task demands [61]. In particular, mounting evidence suggests that the IAPF may serve as an indicator of transient, overarching brain states [37]. The widespread topographical distribution of high SVM weights supports the notion that this effect is indicative of a comprehensive neural state. We, therefore, conjecture that the greater variability in alpha peak center frequency could reflect increased demands on attention processes, while HHSIs engage in greater mental absorption and response preparation than LHSIs during the induction. This increased demand is likely to yield greater dynamics of overall neural states. A recent meta-analysis connects feelings of relaxation to alpha-band activity [62]. However, these effects are observed through variations in the alpha rhythms’ power, not variance in the center frequency, while our recent and current work shows the effects of induction and hypnotic susceptibility do not involve alpha power [24]. Furthermore, evidence has been inconsistent in relating alpha-band power to hypnosis [27]. Future investigations will therefore be necessary to clarify how the IAPF contributes to hypnotic phenomena.

Some limitations curb the scope of the present research. First, the scores from the HGSHS:A approximate a normal distribution in the population [10], indicating that future studies integrating machine learning and multivariate analyses should transition from treating this as a classification problem to a regression problem. This shift would involve predicting actual HGSHS:A scores based solely on EEG recordings. Such a transition, however, would necessitate a considerably larger sample size than that used in the current study. In this manner, our results offer additional evidence for the feasibility of predicting individual scores based on physiological data. Second, our study did not collect data on the phenomenological and cognitive states of participants during hypnosis. Prior research underscores the importance of understanding these states to fully grasp hypnotic phenomena [58,63]. Including cognitive and phenomenological assessments would therefore provide valuable insights for interpreting our findings related to the variability in the IAPF as a function of hypnotic susceptibility and hypnotic induction.

In summary, our findings advance our understanding of the neurophysiological mechanisms of hypnosis by showing that the dynamics of alpha-band activity relate to hypnotic susceptibility and hypnotic induction. Specifically, in contrast to previous research that primarily focused on oscillatory power as an indicator of hypnotic states, we found that the variability in the IAPF represents a central feature of hypnotic susceptibility. Interestingly, in our study, this effect was not related to changes in complexity of the EEG signal. These observations reveal that hypnotic phenomena may be associated with the dynamic adaptability of the brain’s neural rhythms, wherein greater variability in the IAPF is indicative of mental preparation. Future research should continue to explore these dynamic aspects of neural activity, particularly in relation to cognitive and phenomenological changes during hypnosis, to better understand the neural basis of hypnotic experiences and enhance clinical applications of hypnotic techniques.

## Data Availability

Data for the analyses supporting the current study are publicly available on the Open Science Framework repository: https://osf.io/unq6j/, accessed on 25 August 2024. Code for the analyses supporting the current study is publicly available on the Open Science Framework repository: https://osf.io/unq6j/, accessed on 25 August 2024.

## Supplementary Materials

The following supporting information can be downloaded at: https://www.mdpi.com/article/10.3390/brainsci14090883/s1. Section S1: The script for the hypnotic induction.
